# Low-Density Lipoproteins Oxidation and Endometriosis

**DOI:** 10.1155/2013/624540

**Published:** 2013-06-19

**Authors:** Grzegorz Polak, Bartłomiej Barczyński, Wojciech Kwaśniewski, Wiesława Bednarek, Iwona Wertel, Magdalena Derewianka-Polak, Jan Kotarski

**Affiliations:** 1st Department of Oncological Gynecology and Gynecology, Medical University of Lublin, Staszica 16, 20-081 Lublin, Poland

## Abstract

The etiopathogenesis of endometriosis still remains unknown. Recent data provide new valuable information concerning the role of oxidative stress in the pathophysiology of the disease. It has been proved that levels of different lipid peroxidation end products are increased in both peritoneal fluid (PF) and serum of endometriotic patients. We assessed the concentration of oxidized low-density lipoproteins (oxLDL) in PF of 110 women with different stages of endometriosis and 119 women with serous (*n* = 78) or dermoid (*n* = 41) ovarian cysts, as the reference groups. PF oxLDL levels were evaluated by ELISA. We found that concentrations of oxLDL in PF of endometriotic women were significantly higher compared to women with serous but not dermoid ovarian cysts. Interestingly, by analyzing concentrations of oxLDL in women with different stages of the disease, it was noted that they are significantly higher only in the subgroup of patients with stage IV endometriosis as compared to women with ovarian serous cysts. In case of minimal, mild, and moderate disease, PF oxLDL levels were similar to those noted in reference groups. Our results indicate that disrupted oxidative status in the peritoneal cavity of women with endometriosis may play a role in the pathogenesis of advanced stages of the disease.

## 1. Introduction

Despite many years of research efforts and impressive progress in knowledge of mechanisms of endometriosis development, the etiopathogenesis of the disease and exact cause of infertility in patients suffering from endometriosis still remain poorly understood. None of the theories and models of endometriosis pathogenesis provide definitive explanation of the disease development, considering its different manifestations and various localizations. Recently published studies present new data on potential role of free radicals in endometriosis pathophysiology. Although the origin of the oxidative stress occurring in the peritoneal cavity in endometriotic patients is unknown, accumulating data suggest that increased iron levels, together with apoptotic endometrial fragments and activated macrophages, may promote prooxidant environment. In addition, oxidative stress in endometriotic patients may potentially be induced by environmental factors, including dioxins or heavy metals [1, 2].

In our preliminary work we found significantly increased levels of ox-LDL in peritoneal fluid of women with stage III/IV endometriosis compared to patients with follicle ovarian cysts. However, peritoneal fluid oxLDL concentrations did not differ significantly between patients with minimal/mild endometriosis and women from the reference group [3]. Murphy et al. [4] showed increased low-density lipoprotein (LDL) oxidation in peritoneal fluid of patients with endometriosis, which may be a result of peritoneal cavity macrophages hyperactivity. It was also proved that oxidized LDLs stimulate monocyte chemotactic protein-1 (MCP-1) expression in mesothelial and endometrial cells which provides direct evidence of oxidative stress role in etiopathogenesis of the disease [5]. Increased concentrations of lipid peroxidation end products, malondialdehyde (MDA), 8-isoprostane, and 25-hydroxycholesterol, were found in peritoneal fluid of infertile women with endometriosis [6–10]. Serum of patients with endometriosis, compared to healthy women, contains also significantly higher 8-isoprostane levels [11]. Murphy et al. [12] showed that peritoneal fluid of patients with endometriosis contains increased concentration of lysophosphatidylcholine, another lipid peroxidation product with confirmed chemotactic properties for monocytes. MDA and 7-hydroxynonenal (HNE-7) expression were increased in endometriosis implants tissue; however, both lipid proteins are also expressed in eutopic endometrium [4]. Concentrations of antibodies against lipid peroxidation products were found to be increased in serum of women with endometriosis, with no immunoglobulins detected in their peritoneal fluid [13]. Serum of women with endometriosis contains also elevated concentration of lipid hydroperoxide (LOOH), and its levels correlate positively with the stage of the disease according to revised American Fertility Society classification [14]. Peritoneal fluid of endometriotic patients contains oxidatively modified protein-lipid complexes, showing both chemotactic properties and ability to stimulate selected cytokines production [15]. However, there are relevant data published in the literature according to which the concentrations of MDA and MDA-Cu complexes demonstrate no significant differences, being comparable in peritoneal fluid of patients with and without endometriosis, showing also no significant correlation with the stage of the disease [16–18]. No differences were also found in peritoneal fluid concentration of another lipid peroxidation product, cholest-3,5-dien-7-one [18].

The objective of the study was to assess concentrations of oxidized low-density lipoproteins (oxLDL) in peritoneal fluid (PF) of women with endometriosis.

## 2. Material and Methods 

We examined 229 women, aged 15–53, who underwent diagnostic or therapeutic laparoscopy. Clinically and histologically confirmed diagnosis established the following groups: women with endometriosis (E, *n* = 110) and as the reference groups: patients with simple serous (R1, *n* = 78) and dermoid (R2, *n* = 41) ovarian cysts. In each case, endometriosis was staged according to the American Society for Reproductive Medicine classification [19]. The disease was found to be minimal (E1) in 23 cases, mild (E2) in 25 patients, moderate (E3) in 39 women, and severe (E4) in 23 cases. 

Subjects were not given hormonal therapy and/or anti-inflammatory medications for at least 3 months before laparoscopy. Medical history of the patients and basic clinical examination showed no general chronic diseases, except for the condition, which was the indication for laparoscopy. Mean age of women did not differ significantly between the studied groups. Similarly, no significant difference was found in the phase of menstrual cycle of the time of laparoscopic procedures between women in all study groups.

All patients signed an informed consent, and the Lublin Medical University Ethics Committee approval was obtained for the study.

All visible PFs were aspirated during laparoscopy from the anterior and posterior cul-de-sacs, under direct vision to avoid blood contamination. Samples were immediately centrifuged at 500 ×g for 5 minutes, and the supernatants were aspirated and stored at −70°C until analysis. OxLDL concentration in the PF was measured in duplicate using a commercially available enzyme-linked immunoassay kit (Immundiagnostik AG, Cat. no. K7810). The concentration of oxLDL was expressed in nanograms per milliliter.

 For further analysis all data were tested with the Shapiro-Wilk test for normality. Because data were not normally distributed, statistical significance between E and R groups was determined with the Mann-Whitney *U* test. The Kruskal-Wallis H test was used for comparisons between the E groups. *P* value less than 0.05 was considered statistically significant. Data are presented as medians (Me), minima (Min), maxima (Max), and lower and upper quartiles. 

## 3. Results

**Figure 1:**
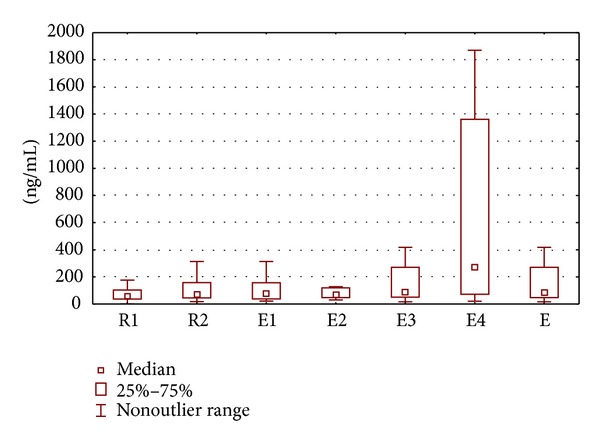
Concentrations of oxLDL in PF of studied women.

Oxidized low-density lipoprotein levels were detectable in all PF samples. Concentrations of oxLDL in PF of patients with endometriosis were significantly higher compared to women with serous ovarian cysts (*P* = 0.03). However, no significant difference in the PF oxLDL levels was found between patients with endometriosis and women with dermoid ovarian cysts (*P* = 0.4). Levels of oxLDL in PF of women with serous ovarian cysts were similar to those noted in patients with dermoid cysts (Figure 1, Table 1).

By analyzing concentrations of oxLDL in PF of women with different stages of the disease, it was noted that they were higher only in the subgroup of patients with stage IV endometriosis as compared to women with ovarian serous cysts. No significant differences were found between concentrations of oxLDL in PF of women with different stages of the disease (Table 2). 

PF oxLDL concentration did not differ significantly between the subgroups of women in the follicular and the luteal phase of the menstrual cycle (Me, range: 71.5, 1.2–1470 ng/mL versus 80.3, 16.7–1870 ng/mL, *P* = 0.5). 

## 4. Discussion

In our work, we demonstrated that peritoneal fluid oxLDL concentration was significantly higher in patients with endometriosis than in women with serous ovarian cysts; however, it did not differ significantly as compared to subjects with dermoid cysts. After analysis of data obtained in women with different stages of the disease, it was noted that these results are found only in patients with severe endometriosis. In case of minimal, mild, and moderate disease, peritoneal fluid oxLDL levels were similar to those noted in reference groups. To our knowledge, only Murphy and colleagues investigated the possible role of oxLDL in the pathogenesis of endometriosis. Based on a small number of cases, they found increased oxidation of low-density lipoprotein in women with pelvic endometriosis and increased levels of oxLDL in the PF of patients with this disease [20]. Our results agree with these findings. However, our data suggest that only the severe stage of endometriosis is associated with increased oxidation of low-density lipoprotein in the peritoneal cavity, probably as the result of an imbalance in prooxidant/antioxidant PF systems.

Shanti et al. [13] demonstrated that women with endometriosis had increased serum concentrations of autoantibodies to markers of oxidative stress including oxLDL. Data from our work indirectly confirm these results. Based on many findings, there is an emerging concept of treating endometriosis as an autoimmune disease. An increased incidence of endometriosis was observed in the group of women with autoimmune diseases such as multiple sclerosis, lupus erythematosus, psoriasis, Crohn's disease, hypothyroidism, hyperthyroidism, and rheumatoid arthritis. Endometriosis shares many similarities with other autoimmune diseases including elevated levels of cytokines, decreased cell apoptosis, and T- and B-cell abnormalities. A variety of autoantibodies have been detected in patients with endometriosis patients, which suggests a polyclonal activation of B cells. The most commonly reported types are antiendometrial, antiovarian antibodies, and autoantibodies against phospholipids, histones, and nucleotides. Other similarities between endometriosis and autoimmune diseases include familial occurrence, tissue damage, preponderance of females, and multiorgan involvement [21, 22]. Lipid peroxidation processes are closely associated with the pathophysiology of autoimmune diseases. Therefore, increased levels of oxidized LDL in PF of women with endometriosis support the theory of treating endometriosis as an autoimmune disease.

Oxidized LDL induces secretion of numerous proinflammatory cytokines including macrophage colony-stimulating factor (M-CSF), interleukin-6 (IL-6), and tumor necrosis factor *α* (TNF-*α*) [23]. Concentrations of these cytokines were found to be elevated in the PF of patients with endometriosis. We can speculate that increased levels of oxidized LDL in the peritoneal cavity of women with severe endometriosis may be one of the factors responsible for increased levels of M-CSF, IL-6, and TNF-*α* in PF. Elevated PF concentrations of these cytokines promote adhesion, invasion, proliferation, and angiogenesis of ectopic endometrium and create an inflammatory environment in the peritoneal cavity in women with endometriosis. Rong et al. [5] demonstrated that oxLDL caused an increase in accumulation of monocyte chemotactic factor-1 (MCP-1) in the medium of cultured mesothelial and endometrial cells. They also found that cells cultured in the presence of PF from endometriosis patients secreted more MCP-1 than those cultured with PF from subjects without the disease. Therefore, we hypothesize that increased concentration of oxLDL may be responsible for, as demonstrated in other studies, higher concentrations of MCP-1 in the PF of women with endometriosis. Stimulation of MCP-1 production by increased PF levels of oxidized LDL may hypothetically be another factor responsible for the creation of proinflammatory environment in the peritoneal cavity of patients with endometriosis. 

Unfavorable changes in lipid profile are present not only in the PF of women with endometriosis. It has been recently shown that plasma of patients with this disease contains higher concentrations of total cholesterol, low-density lipoproteins, high-density lipoproteins (HDL) and triglycerides as compared to healthy women. Although all lipoproteins were significantly elevated in endometriosis patients, the difference was most substantial for LDL levels, which were 38% higher in women with endometriosis [24]. Verit et al. [14] found that patients with endometriosis displayed significantly lower serum levels of HDL and higher levels of triglycerides, total cholesterol, and LDL than women without the disease. They also demonstrated that serum of women with endometriosis is characterized by significantly lower activity of paraoxonase-1 (PON-1), which negatively correlated with the progression of the disease [14]. This HDL-associated antioxidant enzyme with paraoxonase activity prevents LDL and HDL oxidation and is also responsible for the antioxidant effect of HDL. Therefore, authors speculated that unfavorable lipid profile combined with lower PON-1 activity in women with endometriosis may contribute to the increased susceptibility for the development of atherosclerosis.

In conclusion, oxLDL levels were significantly higher in the peritoneal fluid of patients with severe endometriosis than in women with serous ovarian cysts. This suggests that disrupted oxidative status in the peritoneal cavity of women with endometriosis may play a role in the etiopathogenesis of the more advanced stages of the disease. However, it cannot be excluded that high oxLDL levels are the result of more oxidant environment in the peritoneal cavity of patients with severe endometriosis than with less advanced disease. Further experiments are required to specify the potential role of oxLDL in disease progression.

## Figures and Tables

**Table 1:** OxLDL concentrations (ng/mL) in the PF of women with and without endometriosis.

	Me	Min	Max	Lower quartile	Upper quartile
R1	57,4	1,2	1470	36,6	103,7
R2	71,6	17,9	1420	44,8	157,6
E1	75,9	22,1	1134	37,9	157,1
E2	61,6	30,1	1347	47,2	119,4
E3	85,1	16,7	1723	51,4	270,9
E4	270,6	20,8	1870	71,5	1361
E	85,1	16,7	1870	47,3	270,7

**Table 2:** *P* values for comparisons of oxLDL PF concentrations between study groups.

Variable: oxLDL	*P* values for multiple comparisons (two-sided comparisons)					
	The Kruskal-Wallis test					
	R1	R2	E1	E2	E3	E4
R1		1,000000	1,000000	1,000000	1,000000	*0,001582 *
R2	1,000000		1,000000	1,000000	1,000000	0,212814
E1	1,000000	1,000000		1,000000	1,000000	0,147871
E2	1,000000	1,000000	1,000000		1,000000	0,111283
E3	1,000000	1,000000	1,000000	1,000000		0,466813
E4	*0,001582 *	0,212814	0,147871	0,111283	0,466813	
